# Burden of Thyroid Cancer From 1990 to 2019 and Projections of Incidence and Mortality Until 2039 in China: Findings From Global Burden of Disease Study

**DOI:** 10.3389/fendo.2021.738213

**Published:** 2021-10-06

**Authors:** Fang Cheng, Juan Xiao, Chunchun Shao, Fengyan Huang, Lihua Wang, Yanli Ju, Hongying Jia

**Affiliations:** ^1^ Department of Epidemiology and Health Statistics, School of Public Health, Cheeloo College of Medicine, Shandong University, Jinan, China; ^2^ Center of Evidence-Based Medicine, Institute of Medical Sciences, The Second Hospital, Cheeloo College of Medicine, Shandong University, Jinan, China

**Keywords:** thyroid cancer, incidence, mortality, disease burden, projection, China

## Abstract

Projecting the burden of thyroid cancer (TC) over time provides essential information to effectively plan measures for its management and prevention. This research obtained data from the Global Burden of Disease (GBD) Study from between 1990 and 2019 to model how TC will affect China until 2039 by conducting the Bayesian age-period-cohort analysis. The number of new TC cases in China was 10,030 in 1990, 39,080 in 2019, and is projected to be 47,820 in 2039. This corresponds to 3,320, 7,240, and 4,160 deaths, respectively. Disability-adjusted life years (DALYs) cases increased from 103,490 in 1990 to 187,320 in 2019. The age-standardized rate (ASR) of incidence increased from 1.01 to 2.05 during 1990-2019 and was projected to increase to 3.37 per 100,000 person-years until 2039. The ASR of mortality (ASMR) remained stable during the study period and was projected to have a mild decline from 0.39 to 0.29/100,000 during 2020-2039. Although the ASMR in male patients has maintained increasing at a rate of 2.2% per year over the past 30 years, it is expected to decline at a rate of 1.07% per year in 2019-2039. The most significant increase in crude incidence occurred in people aged 45-65 from 1990 to 2019, however, this will shift into young people aged 10-24 from 2020 to 2039. In addition, the proportion of deaths and DALYs caused by obesity increased from 1990 to 2019 and affected men more than women. In conclusion, a substantial increase in counts of incidence of TC in China is projected over the next two decades, combined with the slightly declining mortality, indicating that rational health policies are needed in the future to cope with the increasing number of TC patients, especially among males and adolescents.

## Introduction

Thyroid cancer (TC) is the most prevalent endocrine malignancy (1), accounting for 3–4% of all cancers, with an incidence rate ranking ninth among all cancers in 2020 (2, 3). Approximately 90% of TC are differentiated thyroid cancer (DTC) (4). The World Health Organization histological classification distinguishes four main types of DTC derived from epithelial thyroid cells in which papillary thyroid cancer (PTC) is by far the most frequent form (>85%) (5, 6). Patients with DTC carry an overall excellent prognosis, with appropriate treatment, 5-year survival exceeds 98.3% (7). Most of the known or suspected risk factors for TC are non-modifiable, like patient age, sex, race or ethnicity, and family history (4), while changes in other factors, including obesity, cancer diagnostics, iodine intake, and ionizing radiation, can affect the observed incidence, mortality, and disability adjusted life-years (DALYs) of TC over time (4).

Research based on the Global Burden of Diseases (GBD) study of 2017 have reported an increase in the incidence of TC worldwide (8, 9). However, this incidence varies greatly from country to country, and there is a so-called ‘‘thyroid cancer epidemic’’ across the developed world, in countries such as the USA (10), Canada (11), Australia (12), and Asia (3). The incidence in these countries is more than two-fold higher than in low or middle-income countries (8, 9). Furthermore, even within the same country, the incidence of TC varies significantly. In the 2008-2012 period, the highest incidence in China was in Shanghai, where the age-standardized incidence rate (ASIR) is 22/100,000, 44 times that of Yanting county, the lowest area (13). Additionally, exponential increases are not easy to sustain, a reverse decline or gradual stabilization of incidence has been observed in some countries after 2014 (14, 15).

There were 233,846 new cases and 45,575 deaths associated with TC worldwide in 2019 (8, 9), of which China accounted for 16.71% and 15.88% respectively. China has the highest number of TC cases and deaths in the world (9), and in some provinces (e.g., Zhejiang province) the incidence has dramatically risen to be the highest occurring cancer in female patients (16). Knowing the current landscape and potential trajectories of TC are therefore an important foundation for long-term cancer control actions. Several studies have reported the long-term trend of TC at global and regional levels. We believe this overall trend may not be an accurate reflection of the actual disease burden in China since studies have reported the heterogeneity of TC incidence in different regions (8, 9). Due to the marked alterations in risk factors, further knowledge of future trends in TC incidence and mortality is critical for modification of the national health system to respond to future challenges. Most previous studies, however, are retrospective in nature (3, 10–12) and only one study, based on data from 1982 to 2012 in mainland China, has predicted the incidence of TC by 2032 (17). To address this need, we used a Bayesian age-period-cohort (BAPC) model (18) to analyze the disease burden of TC between 1990 and 2019, and project both future incidence and mortality through to 2039 in China. This will help policymakers assess the burden of thyroid cancer, measure the progress of specific treatments, allocate resources, and formulate relevant policies.

## Materials and Methods

### Data Sources

The GBD Study collected and analyzed data for more than 350 diseases and injuries in 21 regions and 195 countries, covering annual incidence, deaths, DALYs, and risk factors from January 1, 1990, to December 31, 2019. All estimates were generated with 95% uncertainty intervals (95% UIs), which were determined based on the 2.5th and 97.5th-ordered percentiles of 1,000 draws of the uncertainty distribution (19). More detailed information has been published previously (20). In this study, we obtained data on incidence, mortality, DALYs, and related risk factors of TC in China by sex (both genders, male and female) and age (17 age groups, from<5 to ≥80 years at 5-year intervals) for the period 1990–2019 from the GBD Study 2019. This data was extracted using the Global Health Data Exchange query tool (http://ghdx.healthdata.org/gbd-results-tool) (21). For the prediction of TC incidence and mortality, we retrieved the corresponding population data, stratified by year (from 1990 to 2039), sex, and age (17 age groups, from<5years to ≥80 years at 5-year intervals) from the United Nations Department of Economics and Social Affairs Population Division (https://population.un.org/wpp/Download/Standard/Population/). Data analysis was completed on April 5, 2021.

The institutional review board of the Second Hospital of Shandong University in Shandong Province, Jinan, China, determined that the study did not need ethical approval because it used publicly available data. This study followed the Guidelines for Accurate and Transparent Health Estimates Reporting (GATHER) for cross-sectional studies (22).

### Statistical Analysis

Cases were divided into 5-year age-group to describe the age specific incidence, mortality, and DALYs rates of TC. The indicator of estimated annual percentage change (EAPC) was used to reflect the temporal trend of these rates. EAPC is a summative and widely used measure for rate trends over specified intervals, which was calculated according to a regression model fitted to the natural logarithm of the rate, namely *ln* (rate) = *α* + *β* × (calendar year) + **ε**, and EAPC was defined as 100 × (exp (*β*) −1). Its 95% confidence interval (95% *CI*) was also obtained from the linear regression model (23). The rate was deemed to be increased if the EAPC estimation and the lower boundary of its 95% *CI* were both >0. In contrast, the rate deemed to be decreased if the EAPC estimation and the upper boundary of its 95% *CI* were both <0. Otherwise, the rate was deemed to be stable over time.

Previously, several methods have been used to predict cancer incidence based on population data, including the age-period-cohort (APC) model, Nordpred model, Bayesian age-period-cohort (BAPC) model, Bayesian age-period-cohort modeling, and prediction (BAMP) model, and Poisson regression (23–26). Comparative studies based on these methods have demonstrated that age-period-cohort approaches have better predictive performance than time series approaches, especially, the probabilistic forecasts obtained by the Bayesian APC model are well calibrated and not too wide (27, 28).

To further verify the applicability of each method used in the present study, we conducted an additional model comparison study. TC case data from the total population, including male and female patients, were split into training sets (data between 1990 and 2014) and testing sets (data between 2015 and 2019), for training and testing these predictive models, respectively. The absolute prediction error rate was applied to assess the model performance, which was calculated as |(ŷ-y)|/y, where ŷ and y denote the prediction values and the observational values, respectively (25, 26, 28, 29). Since the BAPC model has a relatively low error rate (shown in **Figure S1**), we used it for the projections of TC cases number and rates through 2039.

**Figure 1:**
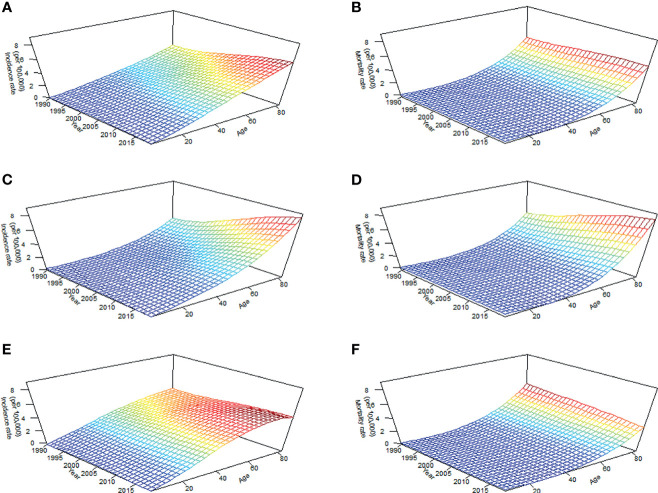
Long-term trends in crude incidence and mortality rate of thyroid cancer by age from 1990 to 2019 in China: incidence **(A)** and mortality **(B)** in total population; incidence **(C)** and mortality **(D)** in men; incidence **(E)** and mortality **(F)** in women.

The BAPC package in the R program (Version 4.0.3; R core team, R Foundation for Statistical Computing, Vienna, Austria) uses integrated nested Laplace approximations (INLA) for full Bayesian inference, which can project mortality or disease rates well (30). In this study, to better determine the APC effects of TC, we plotted long-term trends in morbidity and mortality in **Figure 1**, the effect of age was significant since the incidence and mortality increased with age, and the period effect of incidence changed in the study period, particularly in groups aged over 70 years (**Figure 1A**), while period effects of mortality in all age groups were generally stable through 1990 to 2019 (**Figure 1B**). Furthermore, inconsistent long-term trends for morbidity (**Figures 1C, D**) and mortality (**Figures 1E, F**) were founded between genders. Based on the above information, the second-order random walk (RW2) model with inverse gamma prior distribution was used to analyze age, period, and cohort effects. The grid factor is set to five since the periods are given annually but age groups are given for 5-year groups. In addition, the parameters for the prior distribution for the precision parameter were set as *α*=0.5, 1, and 1 and *λ*=0.0005, 0.00005, and 0.00005 for age, period, and cohort effects, respectively. The World Health Organization (WHO) World Standard Population Distribution (2000−2025) (31) was used to calculate Age-standardized rates (ASRs) per 100,000 person-years through 2039. All data analysis and visualization are completed by R software. A two-sided *P* value of less than 0.05 were deemed statistically significant.

## Results

### Disease Burden in 2019

**Figure 2:**
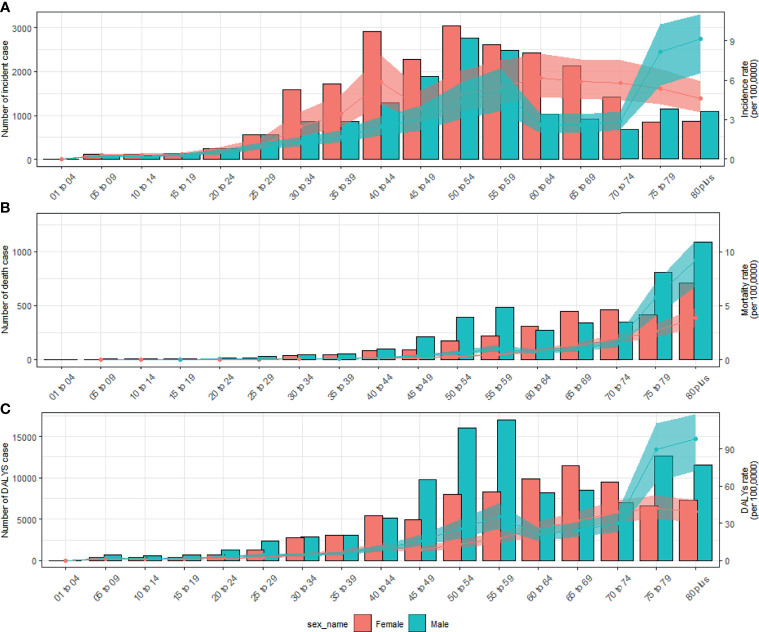
Numbers and rates of incidence **(A)**, death **(B)**, and DALYs **(C)** of thyroid cancer by age and sex in 2019 in China. Shading represents the upper and lower limits of the 95% uncertainty intervals (95% UIs). DALYs, disability-adjusted life-years.

In 2019, the number of newly diagnosed TC was 39,079 cases with an age standard incidence rate (ASIR) of 2.05/100,000 (**Table 1**), which contributed to 7,240 deaths with an age standard mortality rate (ASMR) of 0.39/100,000 (**Table 2**). This caused a further 187,320 DALYs with an age standard DALYs rate of 9.70 per 100,000 person-years (**Table 3**). The highest age specific cases and crude incidence rate were observed in patients aged 50-54 years and 75-79 years, respectively, while both the number of deaths (1,800 cases) and mortality (5.95/100,000) peaked in the ≥80 age group. The 65-69 age group had the largest cases of DALYs with the highest DALYs rate in the 75-79 age group. By genders, the peak of incidence among males and females were ≥ 80 and in the 40-44 age group, respectively (**Figure 2A**). The age specific mortality rates were higher for male than female patients in all age groups except those aged 55-74 years (**Figure 2B**). Similar to death, male patients had a higher number of cases and age standard rate of DALYs, and the group aged over 80 years had the highest DALYs rate in both genders (**Figure 2C**).

**Table 1:** The number and incidence rates of thyroid cancer in China by sex and age, during 1990-2019, and projected until 2039.

	Case number (x 10^3)			Incidence rate (/10^5)			EAPC	
	1990 (95%UI)	2019 (95%UI)	2039 (95%UI)	1990 (95%UI)	2019 (95%UI)	2039 (95%UI)	1990-2019 (95%CI)	2020-2039 (95%CI)
Both^*^	10.03 (8.40,11.91)	39.08 (32.28,47.66)	47.82 (0,134.11)	1.01 (0.86,1.21)	2.05 (1.70,2.50)	3.37 (0,9.45)	2.73 (2.58-2.88)	2.30 (2.28,2.31)
Male^*^	2.51 (2.04,3.10)	16.11 (12.08,20.19)	22.74 (0,67.32)	0.56 (0.445,0.67)	1.74 (1.32,2.16)	3.08 (0,9.11)	4.99 (4.59-5.39)	2.77 (2.75,2.79)
Female*	7.52 (5.91,9.31)	22.97 (17.46,30.16)	25.40 (0,57.01)	1.50 (1.19,1.86)	2.41 (1.83,3.17)	3.56 (0,7.80)	1.57 (1.45-1.68)	1.82 (1.79,1.84)
Age (years)**								
0 to 4	0	0	0.00 (0.00,0.00)	0	0	0.00 (0.00,0.00)	0	0
5 to 9	0.14 (0.09,0.17)	0.21 (0.17,0.25)	0.00 (0.00,0.00)	0.13 (0.09,0.16)	0.29 (0.23,0.34)	0.47 (0.11,2.00)	2.63 (2.07,3.19)	3.35 (3.35,3.35)
10 to 14	0.16 (0.11,0.19)	0.19 (0.16,0.23)	0.42 (0.00,1.17)	0.16 (0.11,0.18)	0.27 (0.23,0.33)	0.52 (0.19,1.48)	1.67 (1.10,2.25)	3.37 (3.36,3.37)
15 to 19	0.22 (0.17,0.27)	0.27 (0.22,0.32)	0.43 (0.00,0.93)	0.17 (0.13,0.21)	0.35 (0.30,0.43)	0.58 (0.29,1.17)	2.27 (2.13,2.42)	3.33 (3.30,3.36)
20 to 24	0.35 (0.26,0.43)	0.49 (0.39,0.65)	0.48 (0.13,0.83)	0.26 (0.20,0.33)	0.6 (0.48,0.79)	0.91 (0.58,1.41)	2.36 (1.89,2.83)	3.08 (2.97,3.19)
25 to 29	0.50 (0.37,0.64)	1.11 (0.88,0.15)	0.78 (0.42,1.13)	0.46 (0.33,0.58)	1.00 (0.80,1.34)	1.47 (1.06,2.04)	2.41 (1.81,3.05)	2.37 (2.11,2.62)
30 to 34	0.79 (0.54,1.00)	2.45 (1.90,3.24)	1.27 (0.85,1.70)	0.89 (0.62,1.13)	1.90 (1.48,2.51)	2.60 (1.91,3.53)	2.04 (1.45,2.64)	1.38 (1.11,1.65)
35 to 39	1.09 (0.81,1.37)	2.58 (2.04,3.39)	2.15 (1.48,2.83)	1.19 (0.89,1.49)	2.56 (2.02,3.36)	3.18 (2.34,4.31)	2.40 (2.24,2.56)	0.86 (0.73,0.99)
40 to 44	1.22 (0.94,1.53)	4.20 (3.34,5.24)	4.45 (3.09,5.82)	1.81 (1.40,2.27)	4.13 (3.29,5.15)	5.08 (3.74,6.89)	2.95 (2.82,3.07)	1.28 (1.02,1.54)
45 to 49	0.79 (0.64,0.95)	4.17 (3.36,5.22)	5.28 (3.67,6.90)	1.52 (1.23,1.83)	3.44 (2.77,4.30)	5.13 (3.78,6.95)	3.09 (2.78,3.41)	2.21 (1.96,2.46)
50 to 54	1.01 (0.82,1.24)	5.80 (4.67,7.17)	9.06 (6.30,11.81)	2.11 (1.72,2.59)	4.63 (3.73,5.73)	7.25 (5.34,9.81)	3.24 (3.03,3.44)	2.42 (2.23,2.61)
55 to 59	1.09 (0.90,1.30)	5.10 (4.07,6.26)	8.08 (5.62,10.54)	2.5 (2.06,3.00)	5.37 (4.29,6.60)	8.68 (6.40,11.75)	3.60 (3.15,4.06)	1.95 (1.69,2.21)
60 to 64	0.78 (0.65,1.02)	3.44 (2.81,4.24)	6.92 (4.81,9.03)	2.21 (1.83,2.87)	4.39 (3.58,5.40)	7.36 (5.43,9.97)	3.05 (2.72,3.39)	2.22 (1.86,2.58)
65 to 69	0.64 (0.53,0.88)	3.03 (2.50,3.70)	8.57 (5.96,11.18)	2.32 (1.95,3.20)	4.30 (3.54,5.26)	7.79 (5.74,10.54)	2.69 (2.41,2.97)	2.76 (2.44,3.07)
70 to 74	0.49 (0.41,0.70)	2.10 (1.73,2.55)	9.49 (6.60,12.38)	2.57 (2.15,3.72)	4.38 (3.62,5.32)	9.72 (7.17,13.15)	2.24 (1.98,2.50)	3.06 (2.85,3.26)
75 to 79	0.42 (0.36,0.57)	1.99 (1.58,2.33)	8.97 (6.24,11.69)	3.68 (3.16,5.04)	6.67 (5.31,7.81)	14.10 (10.40,19.09)	3.01 (2.61,3.42)	2.81 (2.68,2.95)
80+	0.35 (0.31,0.45)	1.93 (1.56,2.23)	9.82 (6.83,12.81)	4.44 (3.83,5.58)	6.39 (5.16,7.37)	13.91 (10.26,18.83)	1.82 (1.53,2.11)	3.08 (2.96,3.21)

EAPC, estimated annual percentage change; 95% UI, 95% uncertainty interval; 95% CI, 95% confidence interval; *, age-standardized of incidence rate; **, crude incidence rate in each age group.

**Table 2:** The number and mortality rate of thyroid cancer in China by sex and age, during 1990-2019, and projected until 2039.

	Case number (x 10^3)			Mortality rate (/10^5)			EAPC	
	1990 (95%UI)	2019 (95%UI)	2039 (95%UI)	1990 (95%UI)	2019 (95%UI)	2039 (95%UI)	1990-2019 (95%CI)	2020-2039 (95%CI)
Both*	3.32 (2.86,4.13)	7.24 (6.01,8.48)	4.16 (0.90,7.43)	0.42 (0.37,0.53)	0.39 (0.32,0.45)	0.29 (0.06,0.52)	0.06 (-0.01,0.21)	-1.34 (-1.37,-1.30)
Male*	1.23 (1.00,1.52)	4.21 (3.18,5.24)	2.88 (0,6.92)	0.35 (0.29,0.43)	0.52 (0.40,0.64)	0.39 (0.00,0.94)	2.20 (1.86,2.54)	-1.07 (-1.11,-1.03)
Female*	2.09 (1.71,2.68)	3.03 (2.40,3.76)	1.50 (0.48,2.50)	0.50 (0.41,0.63)	0.30 (0.24,0.38)	0.21 (0.01,0.35)	-1.78 (-1.86,-1.71)	-1.82 (-1.92,-1.71)
Age (years)**								
0 to 4	0	0	0.00 (0.00,0.00)	0	0	0.00 (0.00,0.00)	0.00 (0.00,0.00)	0.00 (0.00,0.00)
5 to 9	0.02 (0.02,0.03)	0.01 (0.01,0.01)	0.01 (0.00,0.03)	0.02 (0.02,0.03)	0.01 (0.01,0.02)	0.01 (0.00,0.05)	-1.84 (-2.33,-1.35)	-2.27 (-2.28,2.27)
10 to 14	0.03 (0.02,0.03)	0.01 (0.01,0.01)	0.01 (0.00,0.02)	0.03 (0.02,0.03)	0.02 (0.01,0.02)	0.01 (0.00,0.04)	-2.37 (-2.90,-1.84)	-2.24 (-2.25,2.23)
15 to 19	0.03 (0.03,0.04)	0.01 (0.01,0.02)	0.01 (0.00,0.02)	0.03 (0.02,0.03)	0.02 (0.01,0.02)	0.01 (0.00,0.02)	-1.88 (-2.10,-1.66)	-2.23 (-2.24,-2.21)
20 to 24	0.05 (0.04,0.07)	0.02 (0.02,0.03)	0.01 (0.00,0.02)	0.04 (0.03,0.05)	0.03 (0.02,0.04)	0.02 (0.01,0.03)	-1.82 (-2.28,-1.37)	-2.35 (-2.42,-2.29)
25 to 29	0.07 (0.05,0.08)	0.05 (0.04,0.06)	0.02 (0.01,0.03)	0.06 (0.05,0.08)	0.04 (0.04,0.05)	0.02 (0.02,0.03)	-1.68 (-2.26,-1.10)	-2.71 (-2.83,-2.59)
30 to 34	0.08 (0.06,0.10)	0.08 (0.06,0.09)	0.03 (0.02,0.04)	0.09 (0.07,0.11)	0.06 (0.05,0.07)	0.03 (0.03,0.05)	-2.32 (-2.91,-1.71)	-3.06 (-3.15,-2.97)
35 to 39	0.13 (0.10,0.16)	0.09 (0.08,0.11)	0.04 (0.03,0.06)	0.14 (0.11,0.17)	0.09 (0.08,0.11)	0.05 (0.04,0.06)	-1.94 (-2.16,-1.72)	-2.99 (-3.11,-2.87)
40 to 44	0.17 (0.14,0.21)	0.18 (0.14,0.22)	0.08 (0.06,0.11)	0.25 (0.20,0.31)	0.18 (0.14,0.21)	0.10 (0.08,0.12)	-1.40 (-1.55,-1.25)	-2.47 (-2.66,-2.28)
45 to 49	0.16 (0.13,0.20)	0.3 (0.23,0.37)	0.16 (0.12,0.20)	0.32 (0.26,0.38)	0.25 (0.20,0.31)	0.15 (0.12,0.19)	-0.59 (-0.90,-0.27)	-1.94 (-2.05,-1.84)
50 to 54	0.28 (0.23,0.34)	0.57 (0.44,0.70)	0.36 (0.27,0.44)	0.58 (0.48,0.71)	0.45 (0.35,0.56)	0.29 (0.23,0.35)	-0.62 (-0.75,-0.48)	-2.11 (-2.29,-1.92)
55 to 59	0.39 (0.32,0.48)	0.7 (0.56,0.86)	0.45 (0.35,0.55)	0.90 (0.75,1.10)	0.74 (0.59,0.91)	0.49 (0.40,0.59)	-0.13 (-0.39,0.13)	-2.60 (-2.82,-2.38)
60 to 64	0.35 (0.29,0.46)	0.59 (0.49,0.71)	0.51 (0.40,0.62)	0.99 (0.83,1.30)	0.75 (0.62,0.90)	0.54 (0.44,0.66)	-0.69 (-0.85,-0.52)	-2.02 (-2.46,-1.59)
65 to 69	0.38 (0.32,0.51)	0.79 (0.66,0.94)	0.98 (0.77,1.18)	1.38 (1.16,1.88)	1.12 (0.94,1.34)	0.89 (0.73,1.10)	-0.43 (-0.62,-0.24)	-1.19 (-1.56,-0.82)
70 to 74	0.37 (0.31,0.53)	0.81 (0.69,0.96)	1.62 (1.29,1.95)	1.94 (1.63,2.81)	1.7 (1.44,2.01)	1.67 (1.37,2.04)	-0.26 (-0.47,-0.05)	-0.73 (-0.93,-0.52)
75 to 79	0.39 (0.34,0.55)	1.22 (0.97,1.46)	2.38 (1.90,2.86)	3.45 (2.97,4.79)	4.1 (3.26,4.88)	3.77 (3.09,4.59)	1.43 (1.08,1.77)	-1.10 (-1.21,-0.98)
80+	0.42 (0.36,0.52)	1.8 (1.46,2.07)	4.00 (3.20,4.79)	5.20 (4.54,6.53)	5.95 (4.82,6.85)	5.70 (4.67,6.94)	0.91 (0.66,1.16)	-0.94 (-1.05,-0.84)

EAPC, estimated annual percentage change; 95% UI, 95% uncertainty interval; 95% CI, 95% confidence interval; *, age-standardized of mortality rate; **, crude mortality rate in each age group.

**Table 3:** The number and DALYs rates of thyroid cancer in China by sex and age during1990-2019.

	Case number (x 10^3)		DALYs rate (/10^5)		EAPC
	1990 (95%UI)	2019 (95%UI)	1990 (95%UI)	2019 (95%UI)	1990-2019 (95%CI)
Both*	103.49 (87.96,124.72)	187.32 (156.24,219.11)	10.87 (9.33,13.21)	9.70 (8.11,11.27)	-0.20 (-0.31,0.10)
Male*	38.44 (31.36,46.79)	107.11 (81.45,133.26)	8.36 (6.89,10.21)	11.64 (8.91,14.29)	1.84 (1.56,2.13)
Female*	65.05 (52.36,80.81)	80.21 (64.74,99.49)	13.48 (10.98,16.66)	8.11 (6.54,10.07)	-2.00 (-2.11,-1.89)
Age (years)** 0 to 4	0	0	0	0	0
5 to 9	2.04 (0.14,2.44)	0.99 (0.74,1.18)	1.95 (1.38,2.33)	1.36 (1.02,1.63)	-1.56 (-2.06,-1.06)
10 to 14	2.23 (1.65,2.63)	0.92 (0.75,1.10)	2.17 (1.61,2.56)	1.3 (1.06,1.55)	-2.10 (-2.64,-1.57)
15 to 19	2.43 (1.91,2.87)	1.03 (0.85,1.25)	1.91 (1.50,2.26)	1.37 (1.13,1.67)	-1.54 (-1.75,-1.32)
20 to 24	3.70 (2.80,4.61)	1.89 (1.54,2.39)	2.8 (2.11,3.48)	2.31 (1.88,2.92)	-1.47 (-1.91,-1.02)
25 to 29	4.42 (3.29,5.46)	3.6 (2.97,4.52)	4.01 (2.99,4.96)	3.26 (2.68,4.08)	-1.27 (-1.84,-0.69)
30 to 34	4.97 (3.60,6.19)	5.62 (4.54,7.06)	5.61 (4.07,6.99)	4.36 (3.52,5.47)	-1.71 (-2.30,-1.11)
35 to 39	7.19 (5.59,8.84)	6.13 (5.06,7.43)	7.86 (6.11,9.65)	6.08 (5.02,7.37)	-1.36 (-1.58,-1.15)
40 to 44	8.58 (6.90,10.47)	10.52 (8.49,12.50)	12.76 (10.26,15.57)	10.35 (8.35,12.30)	-0.85 (-1.01,-0.69)
45 to 49	7.23 (5.94,8.68)	14.73 (11.87,17.94)	13.97 (11.49,16.78)	12.14 (9.78,14.79)	-0.24 (-0.55,-0.08)
50 to 54	10.73 (8.95,13.01)	24 (19.02,29.40)	22.45 (18.72,27.22)	19.18 (15.20,23.50)	-0.30 (-0.44,-0.16)
55 to 59	13.13 (10.95,15.87)	25.3 (20.49,30.87)	30.21 (25.20,36.51)	26.67 (21.60,32.55)	0.13 (-0.14,0.40)
60 to 64	10.12 (8.51,13.30)	18.09 (15.05,21.77)	28.57 (24.02,37.53)	23.03 (19.16,27.71)	-0.43 (-0.61,-0.26)
65 to 69	9.06 (7.66,12.33)	19.93 (16.80,23.43)	33.11 (27.97,45.06)	28.31 (23.87,33.28)	-0.24 (-0.43,-0.05)
70 to 74	7.13 (5.99,10.30)	16.49 (13.93,19.36)	37.82 (31.79,54.59)	34.46 (29.10,40.45)	-0.12 (-0.33,0.10)
75 to 79	6.08 (5.23,8.42)	19.21 (15.40,21.79)	53.29 (45.82,73.77)	64.35 (51.38,76.26)	1.49 (1.15,1.84)
80+	4.45 (3.87,5.64)	18.86 (15.40,21.79)	55.74 (48.63,70.53)	62.31 (50.88,72.02)	0.89 (0.63,1.16)

DALYs, disability-adjusted life years; EAPC, estimated annual percentage change; 95% UI, 95% uncertainty interval; 95% CI, 95% confidence interval; *, age-standardized rate; **, crude DALYs rate in each age group.

### Temporal Trends of TC From 1990 to 2019

**Figure 3:**
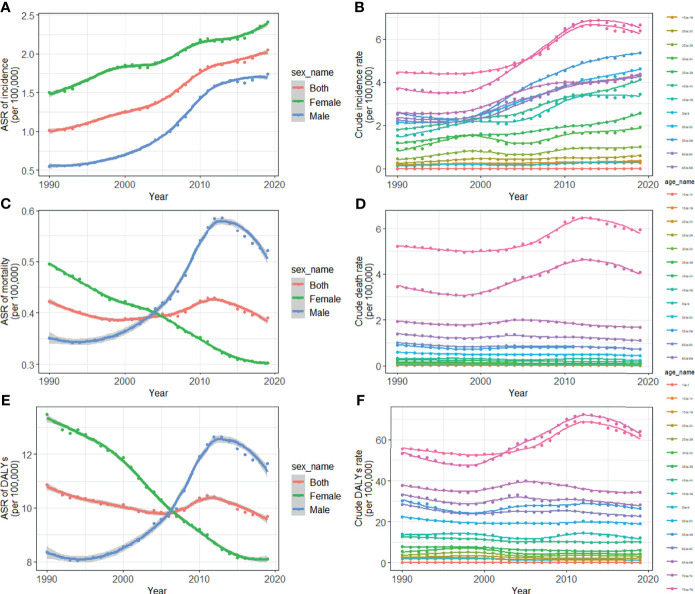
Trends for ASIR **(A)**, age specific incidence rate **(B)**, ASMR **(C)**, age specific mortality rate **(D)**, age-standardized DALY rate **(E)**, and age specific DALYs rate **(F)** of thyroid cancer in China from 1990 to 2019. ASIR, age-standardized incidence rate; ASMR, age-standardized mortality rate; DALYs, disability-adjusted life-years.

TC cases increased from 10,030 in 1990 to 39,080 in 2019, and the ASIR increase nearly doubled with an EAPC of 2.73 (95% *CI*: 2.58, 2.88, **Table 1**). Cancer deaths increased to 7,240 until 2019, but without any statistical significance (EAPC= 0.06, 95% CI: -0.01, 0.21) (**Table 2**). The long-term trend for DALYs is similar to that for death only in terms of numerical variation (EAPC=-0.20, 95% *CI*: -0.31, 0.10) (**Table 3**). The ASIR in female patients was consistently higher than in male patients (**Figure 3A**), while the increased speed of ASIR in male patients with a rate of 4.99% (95% *CI*: 4.59%-5.39%) per year, much higher than female patients of 1.57% (95% *CI*: 1.45%-1.68%). A mildly increasing trend of ASMR (EAPC=2.20, 95% *CI*: 1.86, 2.54) was observed in males, with a rising trend of between 1990 and 2013, which started to decline. In contrast, a decline of ASMR was found among females (EAPC= -1.78, 95% *CI*: -1.86,-1.71), leveling off with males around 2004 (**Figure 3B**). The highest age specific rates of incidence, mortality, and DALYs were observed in patients aged over 75 years from 1990 to 2019 (**Figures 3D–F**).

### TC Deaths and DALYs Attributable to the Risk Factor of High Body-Mass Index (BMI)

In 2019, 8.3% of TC deaths and 9.01% of TC DALYs were attributable to high BMI in male patients. The proportions of male deaths attributable to high BMI increased from 3.72% in 1990 to 8.3% in 2019, and this corresponded to DALYs from 3.63% to 9.01% (**Figures S2A, C**) respectively. The fraction of TC deaths (**Figure S2A**) and DALYs (**Figure S2C**) attributed to obesity was consistently higher in men than in women during the observation period, and the age specific fraction of death (**Figure S2B**) and DALYs (**Figure S2D**) peaked in male patients aged 40-44 years, and in female patients aged 50-54 years.

### Predictions of Incidence and Mortality of TC From 2020 to 2039

**Figure 4:**
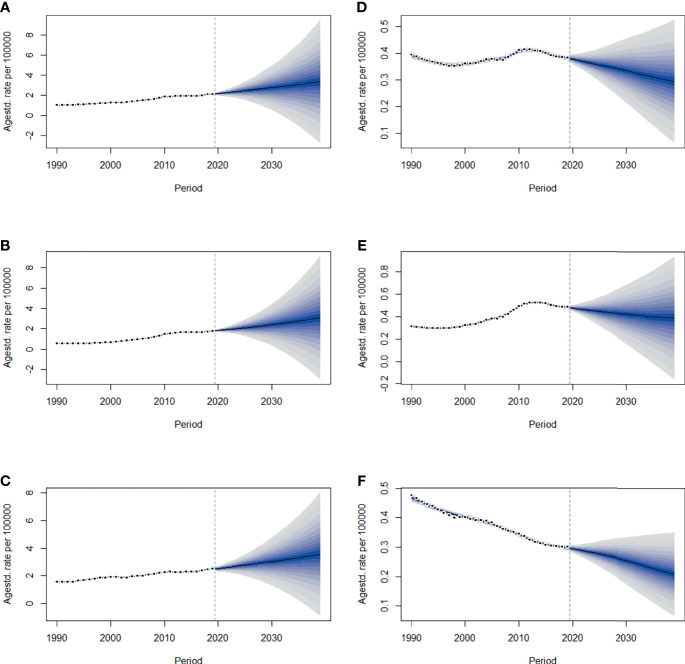
The projections of ASIR **(A–C)** and ASMR **(D–F)** in total population, men and women of thyroid cancer from 2020 to 2039 in China. The open dots represent the observed values, and the fan shape denotes the predictive distribution between the 2.5 and 97.5% quantiles. The predictive mean value is shown as a solid line. The vertical dashed line indicates where the prediction starts. ASIR, age-standardized incidence rate; ASMR, age-standardized mortality rate.

**Figure 5:**
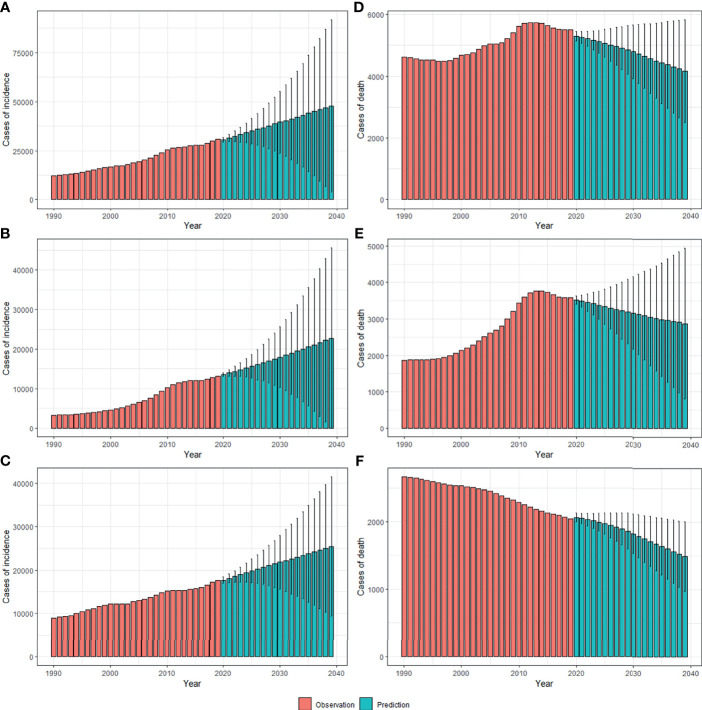
The projections of incidence number **(A–C)** and deaths **(D–F)** in total population, men and women of thyroid cancer from 2020 to 2039 in China. The error bar denotes the 95% credible interval of the predictive value.

By 2039, there will be 47,820 new cases, and the ASIR will rise to 3.37/100,000 with an increasing speed of 2.30% per year through 2020 to 2039 (95% *CI*: 2.28, 2.31) (**Figures 4A**, **5A** and **Table 1**), with a slower growth rate (2.19% per year) compared with 1990 to 2019. The age specific incidence rate will peak in groups aged over 75 years, while adolescents aged 10-24 years old may have the fastest increasing speed with an average EAPC of more than three (**Table 1** and **Figure S3**). The incidence rate in males is expected to reach 3.08/100,000 in 2039 with an EAPC of 2.77 (95%*CI*: 2.75, 2.79), and both the incidence rate (**Figures 4B, C**) and cases (**Figures 5B, C**) will approach that of women. Except for women aged 30-44 years remaining stable, most age groups, both in men and women showed an increasing trend (**Figures S4, S5**).

The mortality rate remained stable from 1990 to 2019, but it is expected that TC mortality and ASMR may gradually decline over the next two decades, with ASMR of TC likely to drop to 0.29/100,000 and deaths to 4,100 by 2039 (**Figures 4D**, **5D**, **Table 1**). The 30-34 age group will see the sharpest decline in mortality rates with an EAPC of -3.06 (95%*CI*: -3.15,-2.97) (**Table 1** and **Figure S6**). Compared with men, ASMR (**Figures 4E, F** and **Table 2**) in women declined more rapidly with fewer deaths (**Figures 5E, F** and **Table 2**) during both the observed and predicted period. Similar to the total population (**Figure S6**), the age specific mortality rate in men will likely see an ongoing decline in people aged under 54 years old and remain stable in those over 55 years old (**Figure S7**). For female patients, the declining trend of mortality rate will be consistent across all age groups (**Figure S8**).

## Discussion

China is one of the three countries with the highest burden of TC in the world (9). The past and the latest disease burden of TC are still unknown. We, therefore, did a comprehensive and in-depth analysis of temporal trends by age, sex, and risk factors, and further applied BAPC models to establish long-term incidence and mortality data and predict TC incidence and mortality in China over the next two decades. Generally, in 2019, the ASR of incidence, mortality, and DALYs of TC were 2.05/100,000, 0.39/100,000, and 9.70/100,000, respectively, far lower than the world average levels (9). From 1990 to 2019, the ASIR of TC increased significantly (about 2.73% per year, on average), ASMR and DALYs, although stable in total population, increased in men and slightly decreased in women. By 2039, the ASIR of TC is projected to reach 3.37/100,000, with a slower growth rate (2.19% per year) compared with 1990 to 2019, while the ASMR of TC is projected to decline to 0.29/100,000 with an EAPC of 1.41% per year.

Compared with the global average increase rate of 1.59% per year (95%*CI*: 1.51, 1.67) (9), China demonstrated the higher speed with an EAPC of 2.05 (95%*CI*: 1.70, 2.50) during the study period. Other research has reported that the peak incidence was aged 50-54 years (32), and while we also found the largest number of cases in this age group, the peak of the incidence was located in those aged 75-79 years old. Consistent with previous research (33), an inverted U –shaped curve of long-term incidence trend was established in the present study, with a peak for women at 40-44 years old and men at older ages. Meanwhile, the incidence of TC is three to four times higher in women than in men. Even though this ratio is consistently observed across countries and remained constant over time (34), in the present study, it fell from 2.68 in 1990 to 1.39 in 2019.

The trend of DALYs has always been consistent with mortality rates. Compared with other countries in the world (35), mortality and DALYs due to TC in China remained relatively stable at low levels throughout the observation period. A recent study based on the Chinese Cancer Registry Annual Report reported a moderate increase in the mortality of TC from 2005 to 2015 (32), and our results agree with this point of view, as we saw a similar increase from 2000 to 2013, as seen in **Figure 2C**, however during the entire observation period of 30 years, this fluctuation is considered to be accidental and not statistically significant. In line with worldwide (9) figures, the peak mortality rate and deaths in China occurred in people aged over 80 years old. Although we saw a slow decline in mortality in females, we observed an increasing trend in males between 1990 and 2019 (EAPC: 2.20, 95%*CI:* 1.86, 2.54).

This situation and the rising incidence of TC accompanied by stable and low mortality may be attributable to improvements in treatment and/or changes in diagnostic practices. The treatment of TC mainly involves surgical resection with or without radioactive iodine therapy, and there has been no substantial change in recent years. However, since the 1990s, the widespread use of ultrasonography and fine-needle aspiration have allowed the detection of papillary thyroid microcarcinoma (PTMC) (tumor size ≤10 mm). Overdiagnosis is the detection and histological confirmation of a disease that would have not been diagnosed in a person’s lifetime had testing not been done (6). A retrospective study of eight cancer registries in China reported the proportion of PTMC was zero in 1972–1985, but 32% in 2000–2014, and the increased incidence of TC was mainly attributed to PTMC (36). PTCM had almost no effect on patients’ life expectancy, correspondingly, a growing body of research suggests that overdiagnosis for PTMC has led to a dramatic increase in TC incidence, which accounted for 87% of all patients with thyroid cancer in China, 77% in the United States, 84% in France, and 90% South Korea (37, 38). Furthermore, South Korea has shown a nearly 15-fold surge in TC incidence in 1993-2011, and a subsequent rapid decrease of 30% after public education on overdiagnosis and overtreatment in 2014 (14). At the same time, after the American Thyroid Association (ATA) clinical practice guidelines first recommended against biopsy of most subcentimeter or small, radiographically low-risk thyroid nodules in 2009, a statistically significant decrease was seen in cancers ≤1 cm from 2013 to 2017 (15). This literature further suggests that the surge in TC incidence should be attributed to overdiagnosis.

In theory, if overdiagnosis was the sole explanation for the increase in TC, the increasing incidence should have been restricted to smaller/localized PTCs, due to the greater effectiveness of treatment for PTCs that are detected early in the disease process, even when it recurs it takes decades for it to cause death, for increased cancer incidence to lead to an increase in mortality a 10–20 year lag should be expected (39). In our study, the ASMR for men increased from 1990 to 2013, which supports the real increase in thyroid cancer incidence. Enewold and colleagues found that although 50% of the increase in PTCs was due to PTMC, tumors greater than 2 cm accounted for 20%, they further reported a 222% increase in the incidence of tumors greater than 5 cm among white women (40). Other investigators have also reported similar results (41), suggesting an increase in the incidence of TC, which leads us to worry whether the “overdiagnosis verdict” is obscuring the real increase in TC, especially among Chinese men. More attention should be paid to male patients with thyroid nodules or who have been exposed to risk factors of TC, in particular the drivers of increasing numbers of advanced-stage PTCs (42), including potential factors and environmental exposure outside of the known influence of radiation (4).

An increase in radiation exposure, particularly from medical sources, has contributed to the increased incidence of PTC (43). However recent studies demonstrate that the prevalence of RET proto-oncogene PTC (RET/PTC) chromosomal aberrations, which are markers of ionizing radiation exposure, have declined in some populations, while point mutations such as BRAFV600E have increased, suggesting that chemical exposure may be more important than ionizing radiation in explaining the increasing incidence (44).

Endocrine-disrupting chemicals (e.g., polychlorinated biphenyls (PCBs), asbestos, pesticides, and polybrominated diphenyl ethers (PBDEs) have been suspected to induce abnormal thyroid cell proliferation, favoring a precancerous state (45). In China, a persistent increase in PBDEs from the 1970s has been reported, with a faster increase since the 1990s (46). PCB concentrations have been found to be increasing rapidly since the late 1950s (peaking in the late 1980s) (47). There is also new evidence to suggest that exposure to specific flame retardants was associated with an increased risk of PTC. The compounds of flame retardants (newer brominated, organophosphate flame retardants) are commonly encountered in household dust (48). Simultaneously, Chinese individuals have also experienced high occupational exposure to benzene and formaldehyde in recent decades (49). Overall, it is likely that changing environmental pollutants due to industrialization in China have played a potential role in TC development.

In addition to environmental pollutants, iodine intake is also an important risk factor. According to the iodine deficiency data obtained from GBD (**Figure S9**), the ASIR of iodine deficiency showed a linear upward trend from 1990 to 2005 and gradually decreased after. Iodized salt might have a lag effect in alleviating iodine deficiency since we know China launched a national iodized salt popularization program in 1996. There is also a lag effect between the incidence of iodine deficiency and TC mortality, and our results confirm that the lag of iodine deficiency over 5 years is highly correlated with TC mortality (Pearsons correlation coefficient=0.85,95%*CI*: 0.69-0.93; *P*<0.001). Iodine deficiency may lead to a highly malignant TC subtype, the correction of iodine deficiency might shift TC subtypes toward less malignant forms (50). Although the causal relationship between iodine intake and BRAF mutation has not been proven, BRAF-positive PTCs were significantly more frequent in Chinese regions with a high iodine intake than in control areas (51). A parallel trend has also been identified between the increase of iodine intake worldwide and the increase in the prevalence of BRAF-positive PTCs (52). However, we must take into account that most of these studies are ecological studies when we explain this phenomenon, which may have ecological fallacies.

Gender differences in TC are always noticeable, over the past 35 years the absolute increase in women was almost four times greater than for men (53). To date, investigations of the biological causes of gender-specific differences in thyroid cancer have been inconclusive (53–55).

Most scholars believe that this difference comes from unequal screening opportunities, since the disparity is mostly confined to the detection of small subclinical PTCs, which are equally common in both sexes at autopsy, and as the lethality of the cancer type increases, the ratio of detection by gender approaches 1:1 (55). However, PMTC is identified during life much more often in women than men. On the one hand, in clinical thinking clinicians have been taught to look for it more often in female patients. On the other hand, there are also differences in patterns of health care utilization. Research shows that women seek and utilize health care more often than men, even when controlling for reproductive health visits (56, 57). Males may be at risk of being diagnosed later because TC was placed lower in the differential diagnosis (55), which may partly explain why male sex has an adverse effect on tumor recurrence and disease-specific mortality of PTC (58–60) and corresponding higher DALYs, despite the low incidence of TC in men.

There are factors specific to women that make them more susceptible to thyroid cancer, such as recent pregnancy, history of infertility, abnormal menstrual cycles, and history of breast cancer (61). In *The Lancet Diabetes and Endocrinology*, Cari Kitahara and colleagues confirmed the significant associations between TC and higher birth weight [odds ratios (OR) per kg 1.14 (95% CI:1.05–1.23)], maternal diabetes before pregnancy [1.69 (0.98–2.93)], and postpartum haemorrhage [1.28 (1.06–1.55)] (62). Meanwhile, thyroid autoimmunity and TC can be concomitant, and studies have identified that thyroid autoimmunity is more frequent in women (63).

Men and women experience different risk factors throughout their lives. A Chinese surveillance study reported that the percentage of drinking and smoking in males is 55.6% and 71.0% respectively, much higher than that of in women (15% and 35.1%) (64), and a health screening study based on a population of four million population indicated that smoking, and possibly alcohol consumption, have true protective effects on the development of TC (65). Furthermore, in our study the proportion of mortality and DALYs caused by obesity increased gradually from 1990 to 2019, and it affected men much more than women. The prevalence of obesity increased by 27.5% in adult individuals and 47.1% in children worldwide from 1980 to 2013 (66), which may be partly responsible for the rising mortality of TC among men. TC causes increased mortality and DALYs in male patients, and the rate of decline in men is much lower than that in women (1.82% in female VS. 1.07% in male patients every year). Thyroid cancer is not a patent for women. More medical screening opportunities should be given to men with related risk factors and thyroid nodules, which can effectively reduce the mortality rate and DALYs caused by TC in males.

Based on the GBD Study 2019, the predicted incidence of TC will keep increasing in the next 20 years for all age groups, and the gender gaps in incidence will further narrow. Age specific incidence rates will increase faster in younger people aged 10-24 years old, with EAPC of over 3%. A similar prediction was reported in a study using data on Cancer Incidence in Five Continents (1983-2032) (17). Of course, if this overdiagnosis is corrected over time, as it is in South Korea (14) and the United States (15), the number of people receiving additional thyroid screening may gradually decrease, then the ASIR of TC could be more conservative than our estimates until 2039. Based on the disease burden trend for women between 1990 and 2019, it may be surmised that in the next two decades, there will be a dramatic increase of TC in men, as incidence is projected to continue to rise and the death rate will level off.

Our study has limitations. First, the predictions were based on the GBD Study, meaning the quality of the original individual registry-based data greatly influences the accuracy and robustness of estimates in the database, and the under-reporting of TC, especially in low income regions, could bias the estimates; however, this bias has been adjusted by mapping the different coding systems to GBD causes. Second, the effects of risk factors were not taken into account in projection models. Similarly, in addition to obesity, the burden of TC attributable to other risk factors was not estimated due to the limited information in the GBD database. Moreover, due to the lack of relevant data, we could not assess the burden and trend of TC stratified by histology, such as PTC and FTC. Finally, the increasingly clear recognition of overdiagnosis worldwide might result in a slight decrease in TC prevalence and consequently bias the predictions in TC incidence. However, using the most up-to-date information and advanced modeling strategies, our study provides a more comprehensive understanding of the TC burden from the past into the future.

In conclusion, even though there will be increased surveillance and overdiagnosis, there is nevertheless truth to the idea that the occurrence of TC will increase, and that there will be a surge in TC patients caused by high morbidity and low mortality, which deserves further investigation. Morbidity in men is increasing faster than in women with a narrowing gap between genders predicted by 2039, combined with higher mortality in men, and the focus of future studies should partly shift from women to men. Efforts are also needed to prioritize TC prevention in younger adults, as the incidence of TC is expected to increase in groups aged 10-24 years.

## Data Availability Statement

Publicly available datasets were analyzed in this study. This data can be found here: (http://ghdx.healthdata.org/gbd-results-tool) and (https://population.un.org/wpp/Download/Standard/Population/).

## Ethics Statement

The institutional review board of the Second Hospital of Shandong University in Shandong Province, Jinan, China, determined that the study did not need approval because it used publicly available data. This study followed the Guidelines for Accurate and Transparent Health Estimates Reporting (GATHER) reporting guideline for cross104 sectional studies.

## Author Contributions

HJ contributed to study design. FC contributed to data analysis, data interpretation, and writing the manuscript. JX and CS contributed to data collection and data analysis. FH and LW revised the manuscript. YJ participated in the preparation of the figures and tables. All authors contributed to the article and approved the submitted version.

## Funding

This project was supported by the Research Development Fund of the Second Hospital of Shandong University (grant no. 11681808), and the 19th batch of Science and Technology Innovation and Development funds of Jinan in 2020 (grant no.202019194).

## Conflict of Interest

The authors declare that the research was conducted in the absence of any commercial or financial relationships that could be construed as a potential conflict of interest.

## Publisher’s Note

All claims expressed in this article are solely those of the authors and do not necessarily represent those of their affiliated organizations, or those of the publisher, the editors and the reviewers. Any product that may be evaluated in this article, or claim that may be made by its manufacturer, is not guaranteed or endorsed by the publisher.
