# Supplementary material for: Burden of Thyroid Cancer From 1990 to 2019 and Projections of Incidence and Mortality Until 2039 in China: Findings From Global Burden of Disease Study
Source: Front Endocrinol (Lausanne). 2021 Oct 6;12:738213. doi: 10.3389/fendo.2021.738213 (PMC8527095; doi:10.3389/fendo.2021.738213)
Supplement: Supplementary file 1 [file DataSheet_1.zip › Supplementary material_revised/Figure S9 Long-term trends in incidence rate of Iodine deficiency by age and sex from 1990 to 2019 in China.pdf]

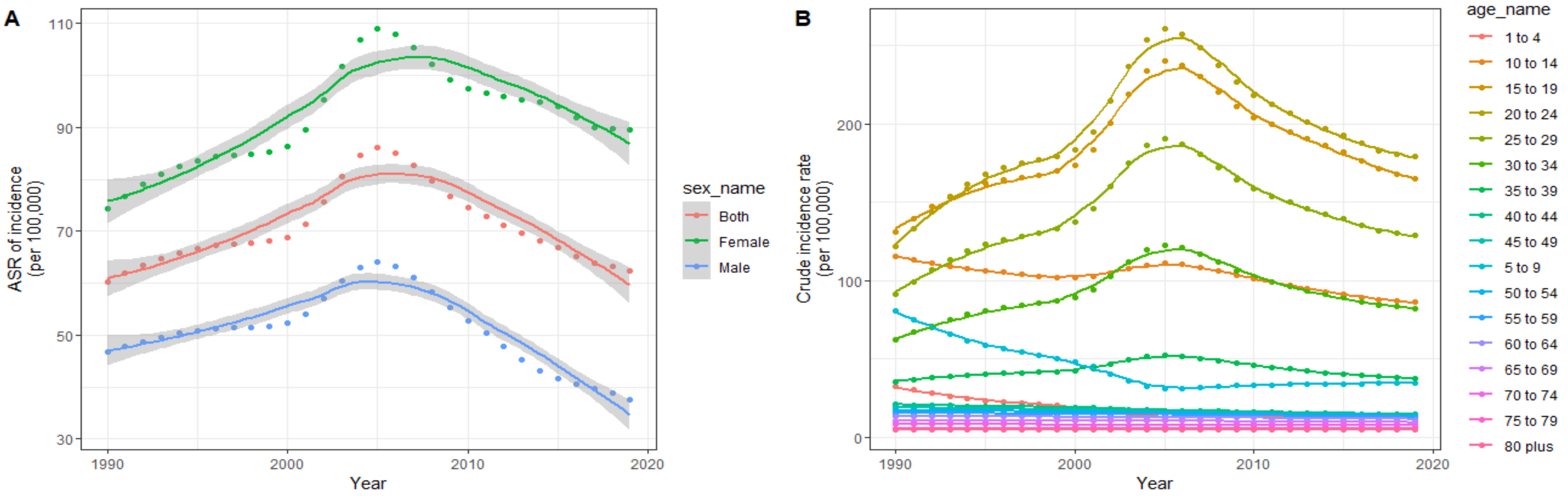

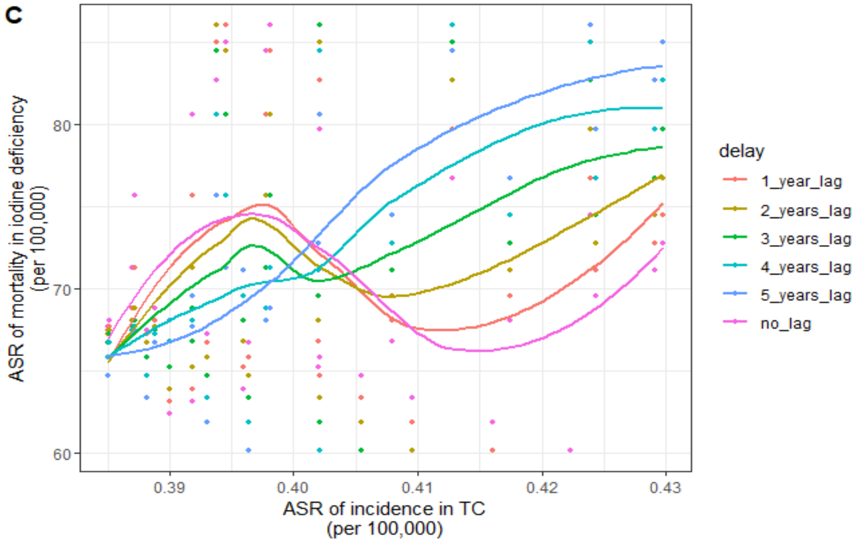

Pearson's correlation between iodine deficiency incidence and TC mortality

Years of lag	*R*	*95%CI of R*	Value of *T*	*P*
No lag	-0.02	(-0.46,0.25)	-0.63	0.53
1 year lag	0.07	(-0.29,0.43)	0.40	0.69
2 years lag	0.28	(-0.10,0.59)	1.50	0.14
3 years lag	0.49	(0.13,0.73)	2.80	<0.01
4 years lag	0.69	(0.41,0.85)	4.63	<0.001
5 years lag	0.85	(0.69,0.93)	7.84	<0.001
