# Supplementary material for: Burden of Thyroid Cancer From 1990 to 2019 and Projections of Incidence and Mortality Until 2039 in China: Findings From Global Burden of Disease Study
Source: Front Endocrinol (Lausanne). 2021 Oct 6;12:738213. doi: 10.3389/fendo.2021.738213 (PMC8527095; doi:10.3389/fendo.2021.738213)
Supplement: Supplementary file 1 [file DataSheet_1.zip › Supplementary material_revised/Supplementary material.docx]

**Figure legends**

Figure S1: The results of model comparison based on ASIR of thyroid cancer in China from 1990 to 2019. APC, age-period-cohort (APC) model; BAMP, Bayesian age-period-cohort modeling and prediction; BAPC, Bayesian age-period-cohort model; Nordpred, Nordpred model; Poisson, Poisson regression model.

Figure S2: Fraction of death and DALYs attributable to high BMI by age and sex in China. Fraction of death (A) and DALYs (C) of thyroid cancer attributable to high BMI by sex from 1990 to 2019 in China, and fraction of death (B) and DALYs (D) of thyroid cancer attributable to high BMI by age and sex in 2019 in China. BMI, body mass index; DALYs, disability-adjusted life-years.

Figure S3: The projections of ASIR by age in total population of thyroid cancer from 2020 to 2039 in China. ASIR, age-standardized incidence rate.

Figure S4: The projections of ASIR by age in men of thyroid cancer from 2020 to 2039 in China. ASIR, age-standardized incidence rate.

Figure S5: The projections of ASIR by age in women of thyroid cancer from 2020 to 2039 in China. ASIR, age-standardized incidence rate; ASMR.

Figure S6: The projections of ASMR by age in total population of thyroid cancer from 2020 to 2039 in China. ASMR, age-standardized mortality rate.

Figure S7: The projections of ASMR by age in men of thyroid cancer from 2020 to 2039 in China. ASMR, age-standardized mortality rate.

Figure S8: The projections of ASMR by age in women of thyroid cancer from 2020 to 2039 in China. ASMR, age-standardized mortality rate.

Figure S9: Long-term trends in incidence rate of Iodine deficiency by age and sex from 1990 to 2019 in China: age standard incidence rate by genders (A) and crude incidence rate by different age groups (B) and scatter plot of mortality from thyroid cancer and incidence of iodine deficiency with different lag periods (C) in total population.
