# Supplementary material for: Burden of Thyroid Cancer From 1990 to 2019 and Projections of Incidence and Mortality Until 2039 in China: Findings From Global Burden of Disease Study
Source: Front Endocrinol (Lausanne). 2021 Oct 6;12:738213. doi: 10.3389/fendo.2021.738213 (PMC8527095; doi:10.3389/fendo.2021.738213)
Supplement: Supplementary file 1 [file DataSheet_1.zip › Supplementary material_revised/figure S6 death rate of different age groups in both genders .pdf]

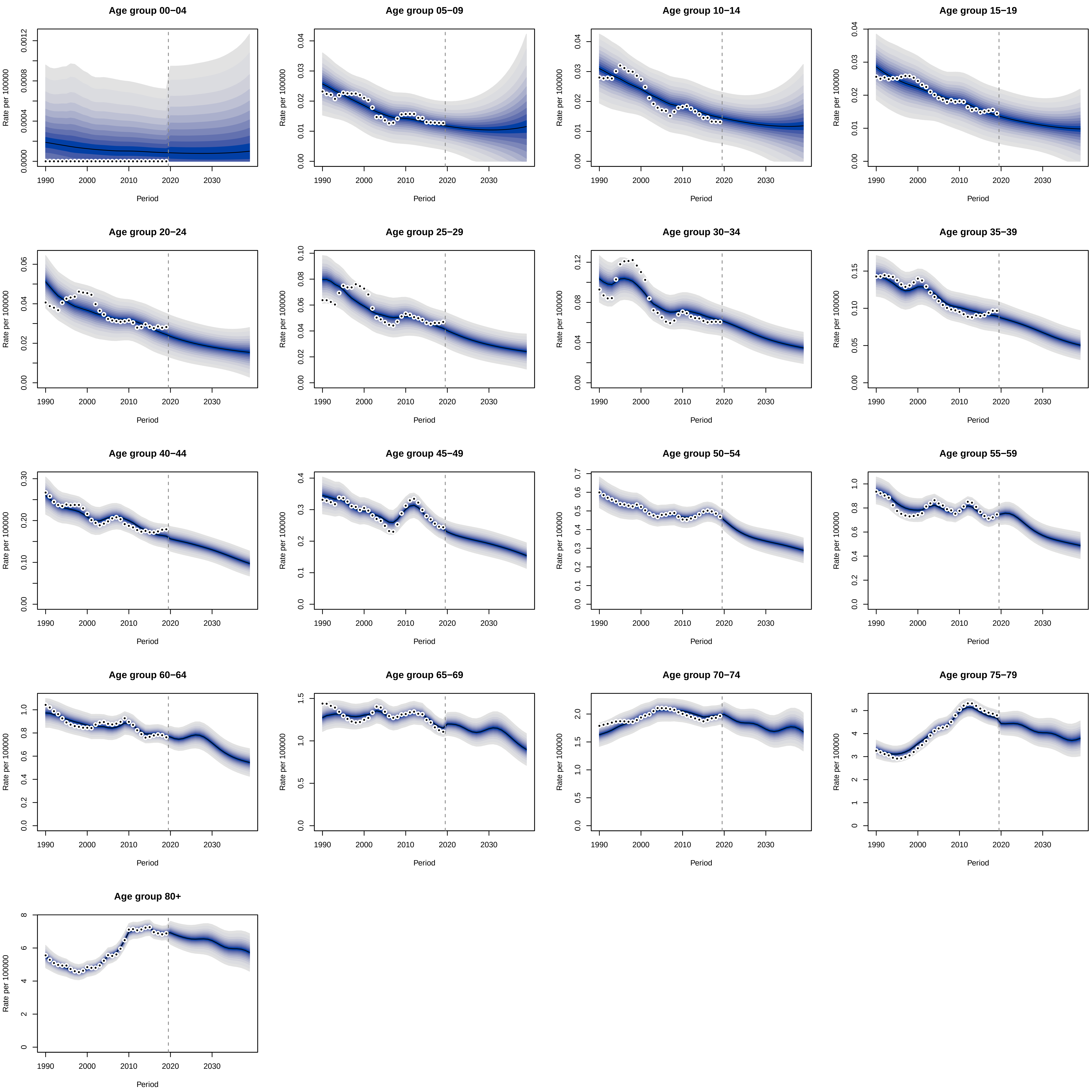
Age group 00–04
Age group 05–09
Age group 10–14
Age group 15–19
Age group 20–24
Age group 25–29
Age group 30–34
Age group 35–39
Age group 40–44
Age group 45–49
Age group 50–54
Age group 55–59
Age group 60–64
Age group 65–69
Age group 70–74
Age group 75–79
Age group 80+
Rate per 100000
Period
1990
2000
2010
2020
2030
