# Supplementary material for: Burden of Thyroid Cancer From 1990 to 2019 and Projections of Incidence and Mortality Until 2039 in China: Findings From Global Burden of Disease Study
Source: Front Endocrinol (Lausanne). 2021 Oct 6;12:738213. doi: 10.3389/fendo.2021.738213 (PMC8527095; doi:10.3389/fendo.2021.738213)
Supplement: Supplementary file 1 [file DataSheet_1.zip › Supplementary material_revised/figure S8 death rate of different age groups infemales .pdf]

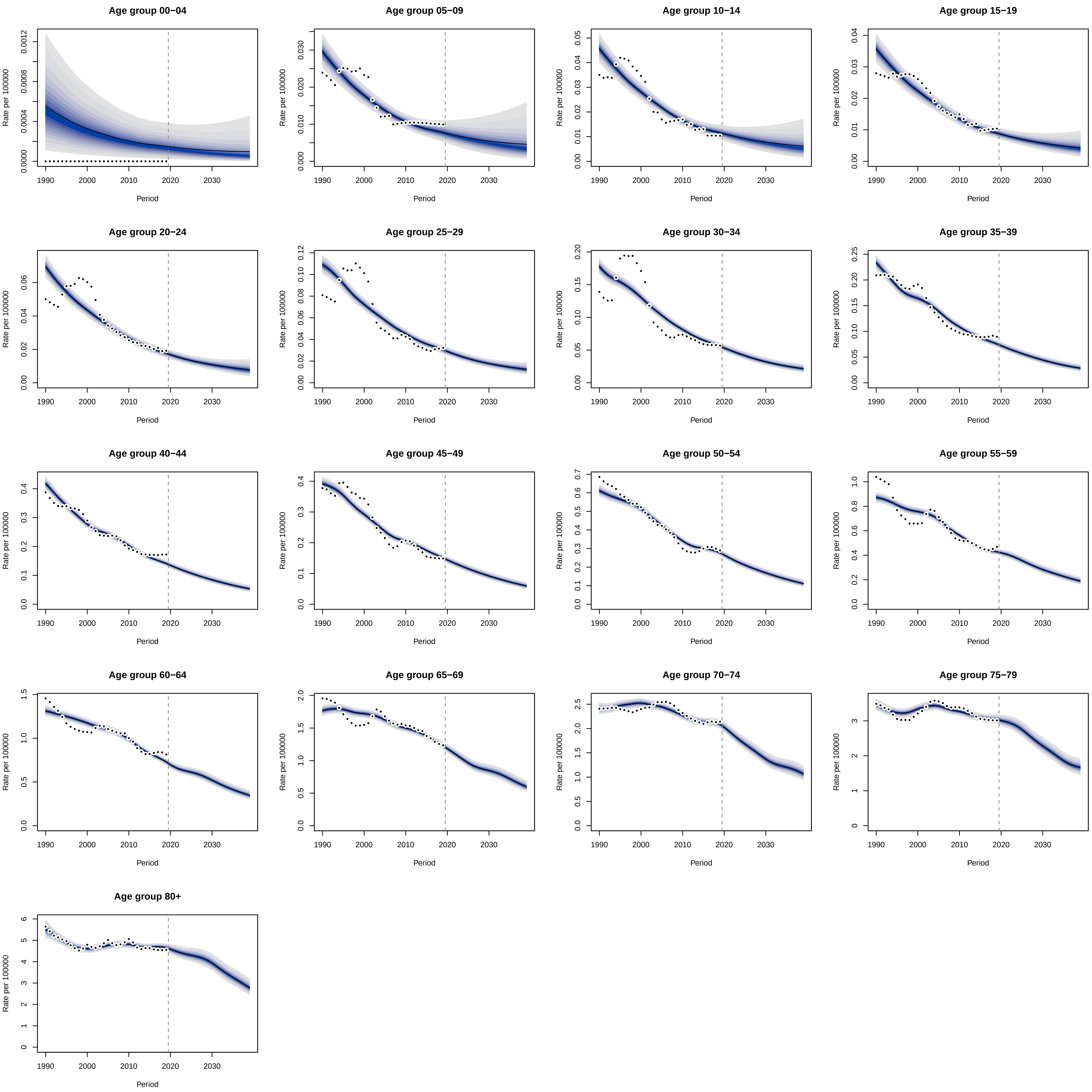
Age group 00−04
Age group 05−09
Age group 10−14
Age group 15−19
Age group 20−24
Age group 25−29
Age group 30−34
Age group 35−39
Age group 40−44
Age group 45−49
Age group 50−54
Age group 55−59
Age group 60−64
Age group 65−69
Age group 70−74
Age group 75−79
Age group 80+
Rate per 100000
Period
1990
2000
2010
2020
2030
