# Supplementary figures and images for: Burden of Thyroid Cancer From 1990 to 2019 and Projections of Incidence and Mortality Until 2039 in China: Findings From Global Burden of Disease Study
Source: Front Endocrinol (Lausanne). 2021 Oct 6;12:738213. doi: 10.3389/fendo.2021.738213 (PMC8527095; doi:10.3389/fendo.2021.738213)

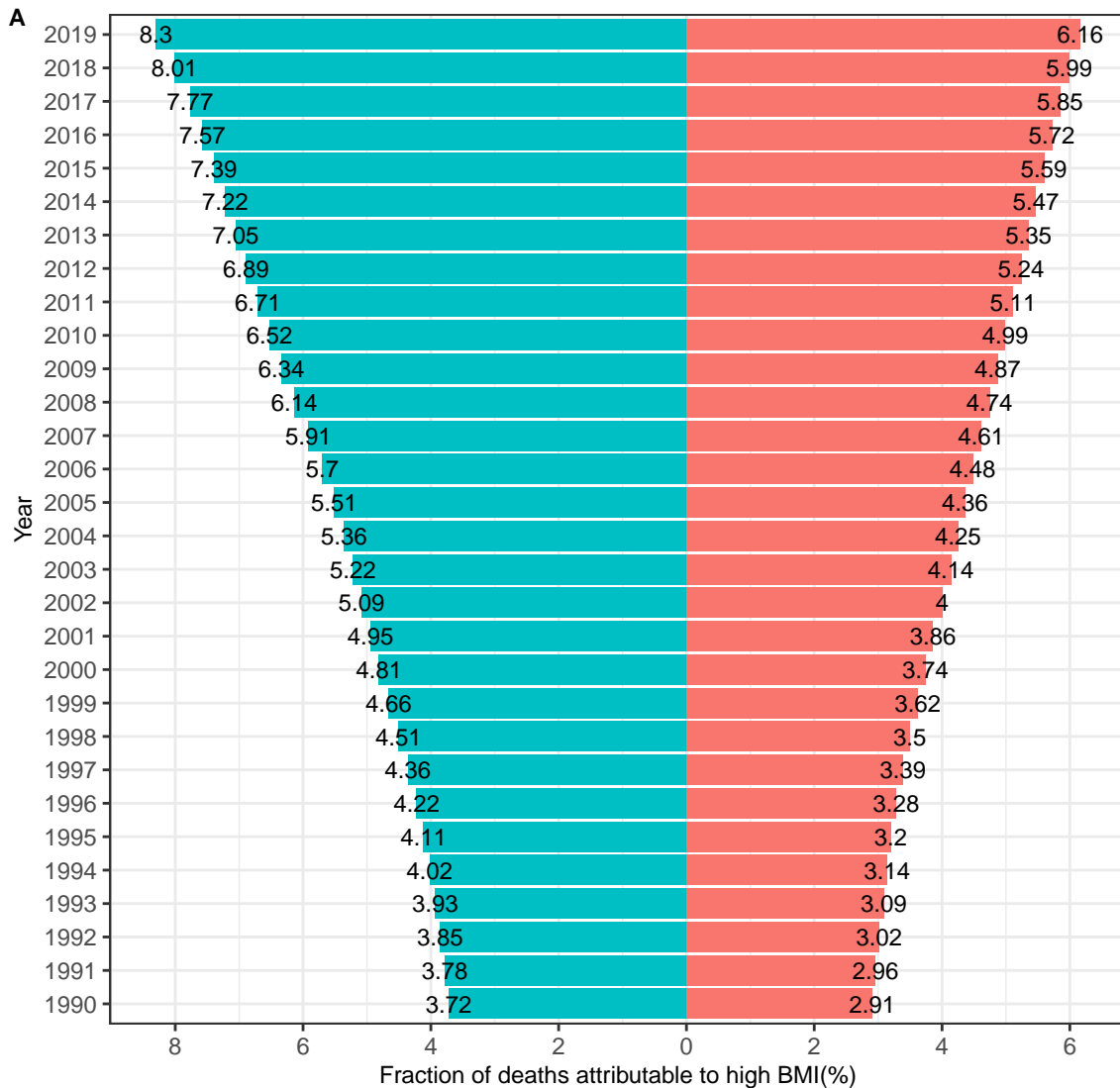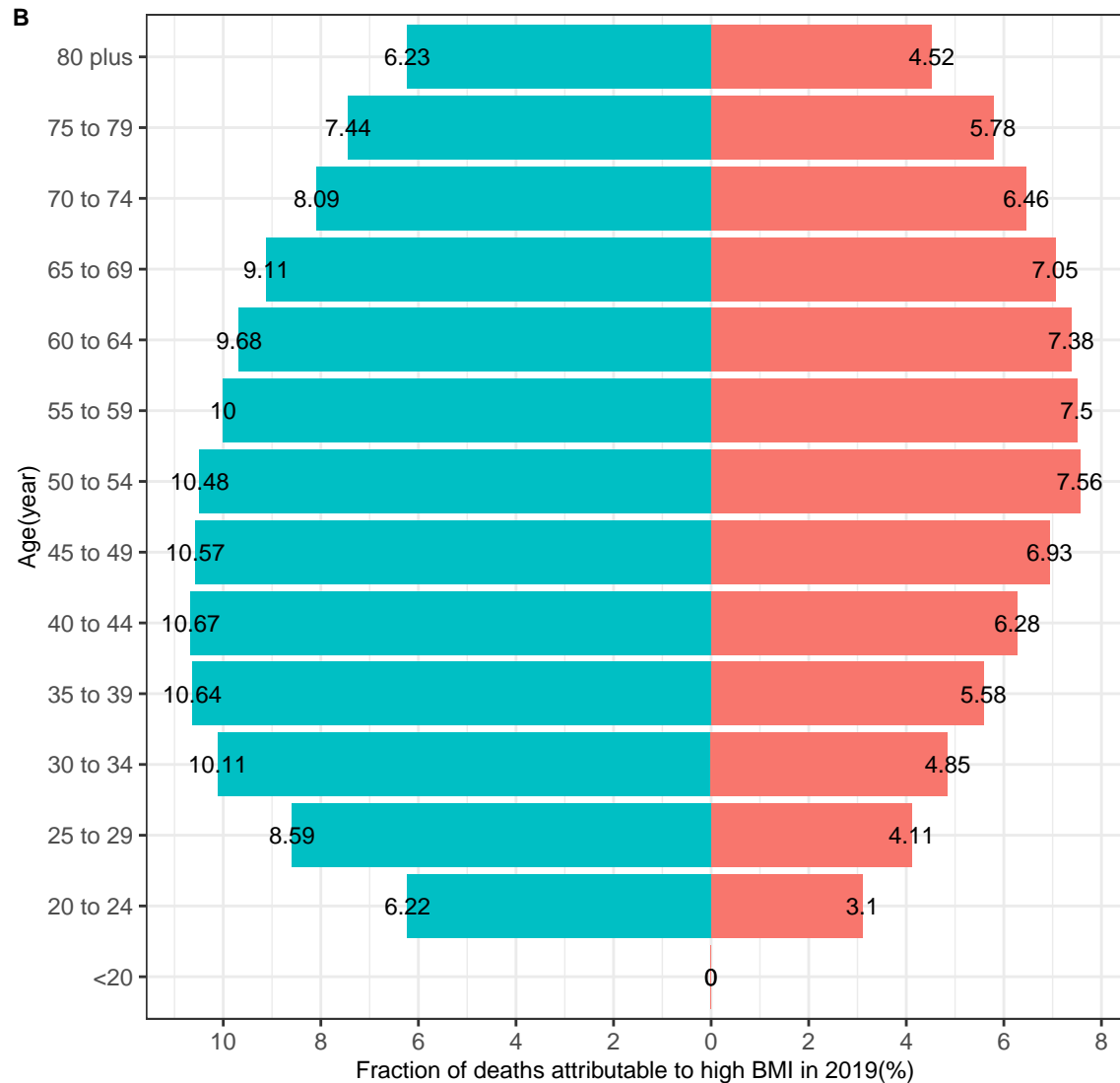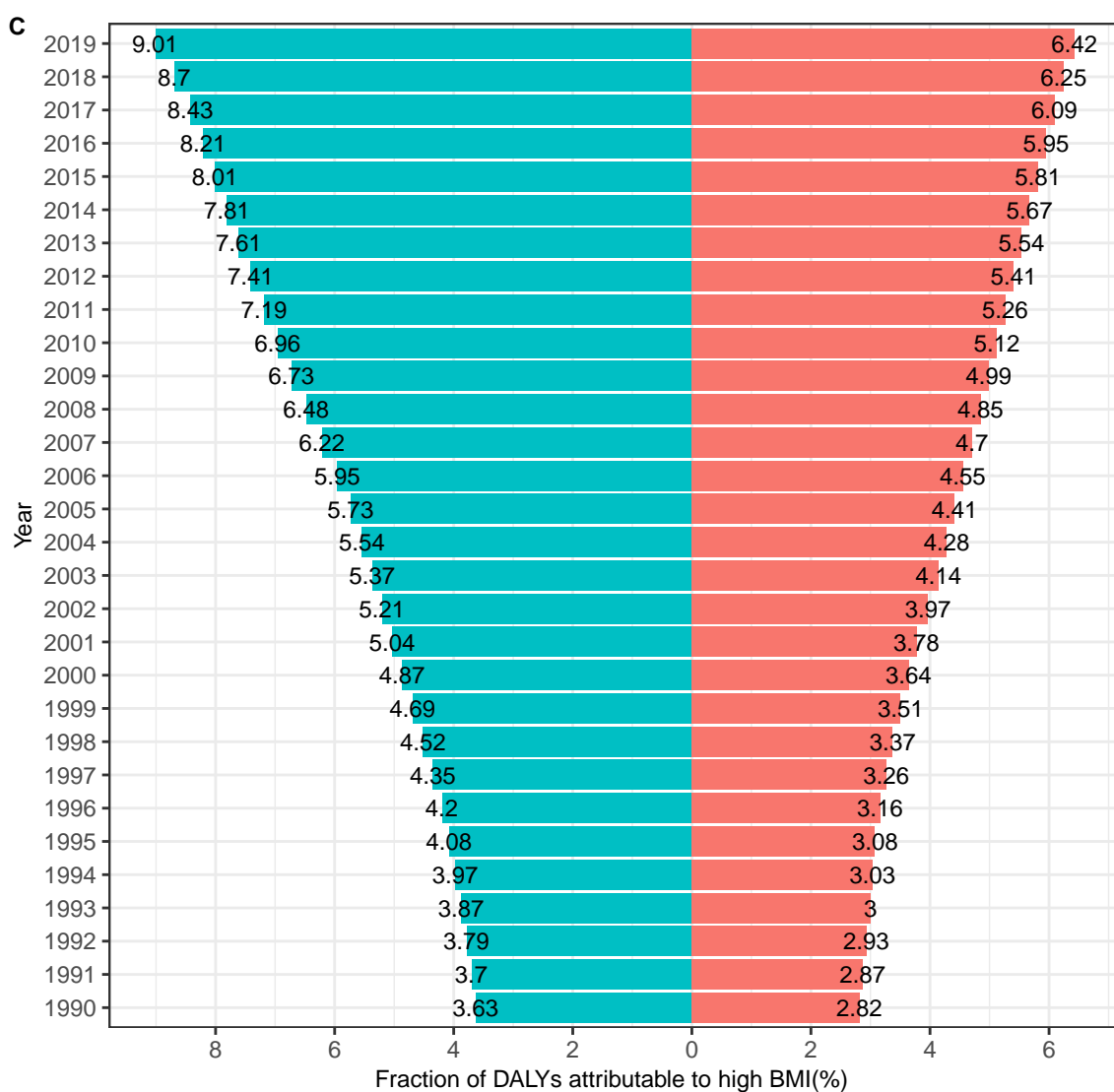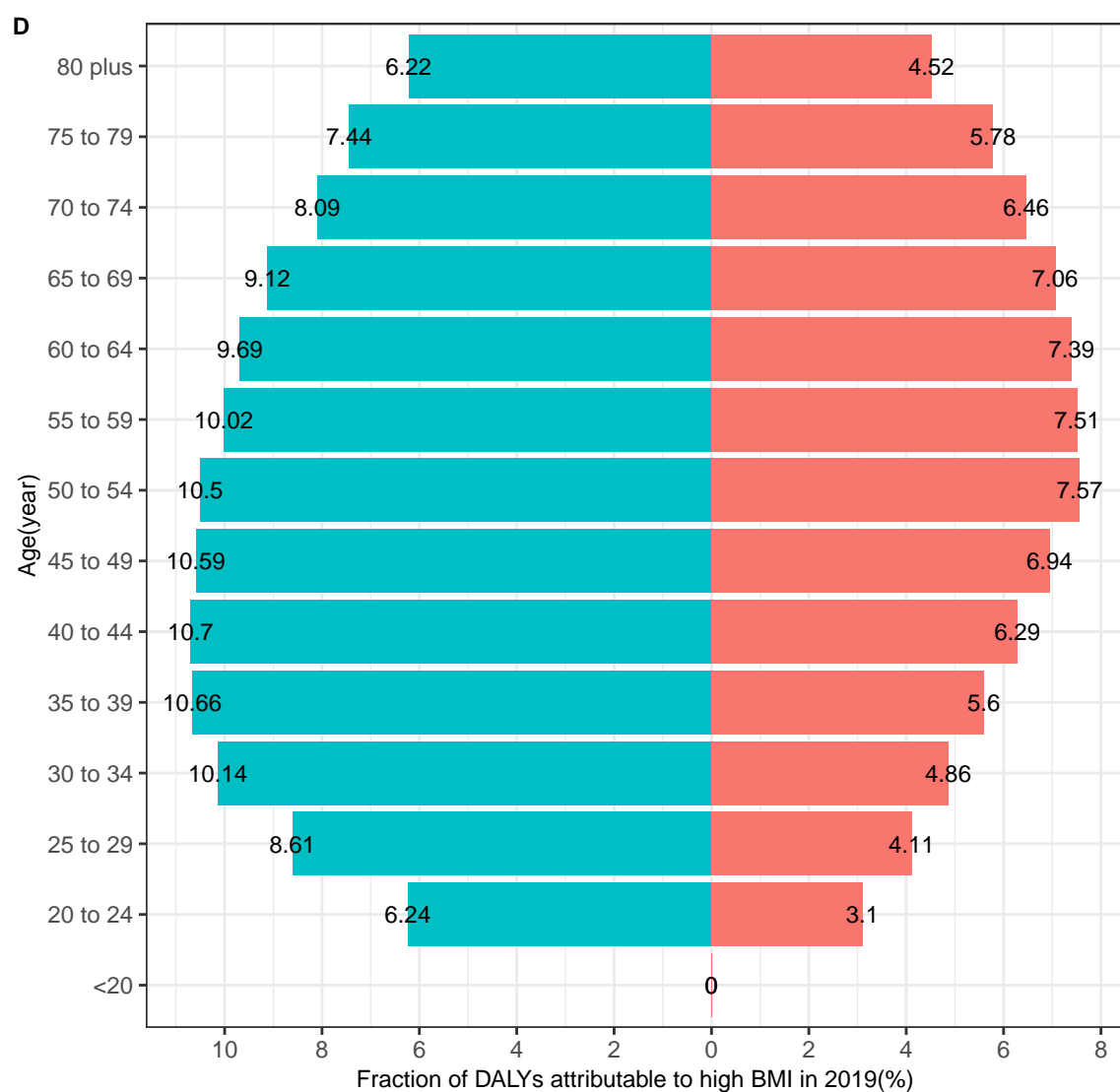

sex\_name Female Male

Supplement: Supplementary file 1 [file DataSheet_1.zip › Supplementary material_revised/Figure S2 risk factor of high BMI.pdf]

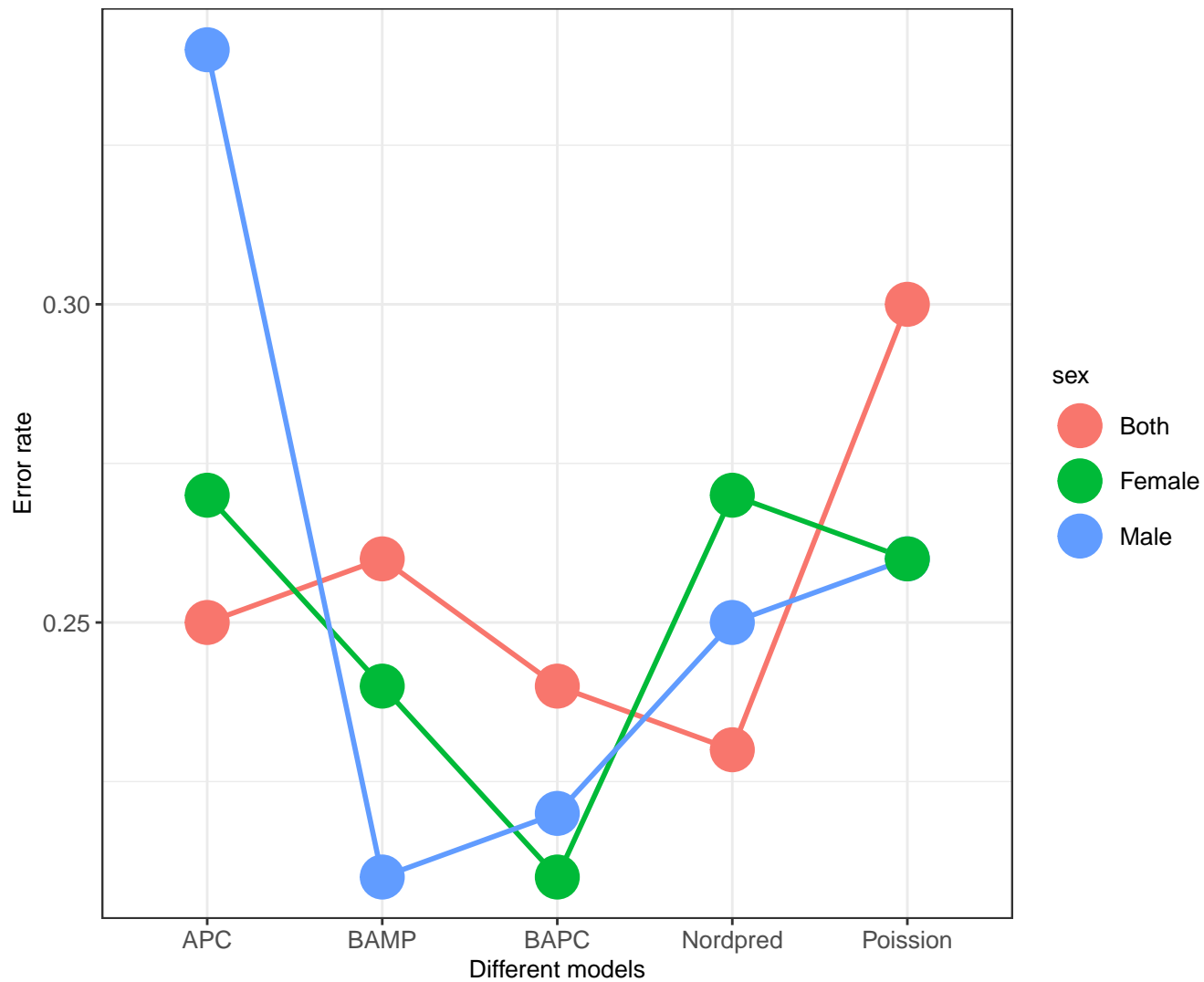

Supplement: Supplementary file 1 [file DataSheet_1.zip › Supplementary material_revised/figure S1 copmarision of several models.pdf]

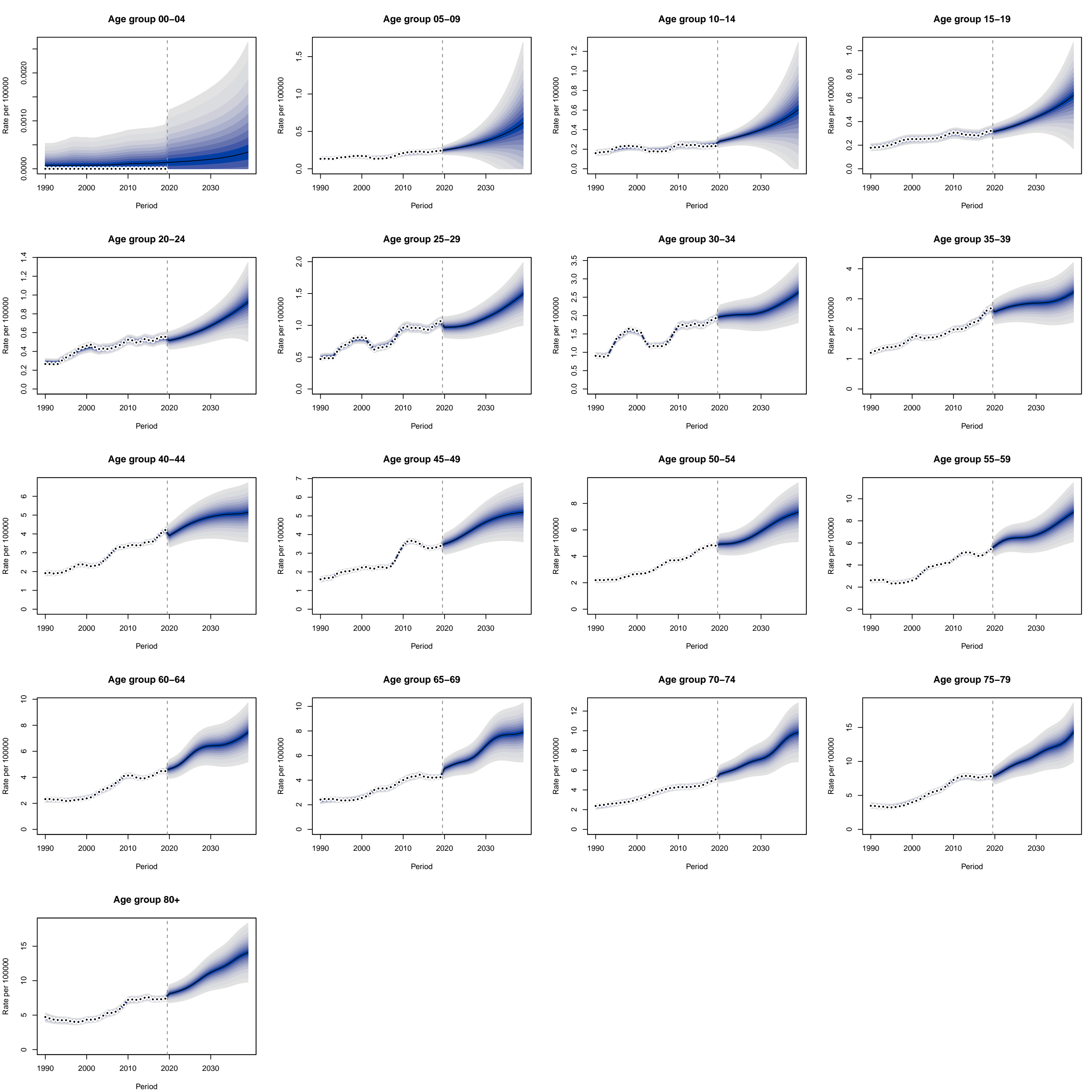

Supplement: Supplementary file 1 [file DataSheet_1.zip › Supplementary material_revised/figure S3 incidence rate of different age groups in both genders .pdf]

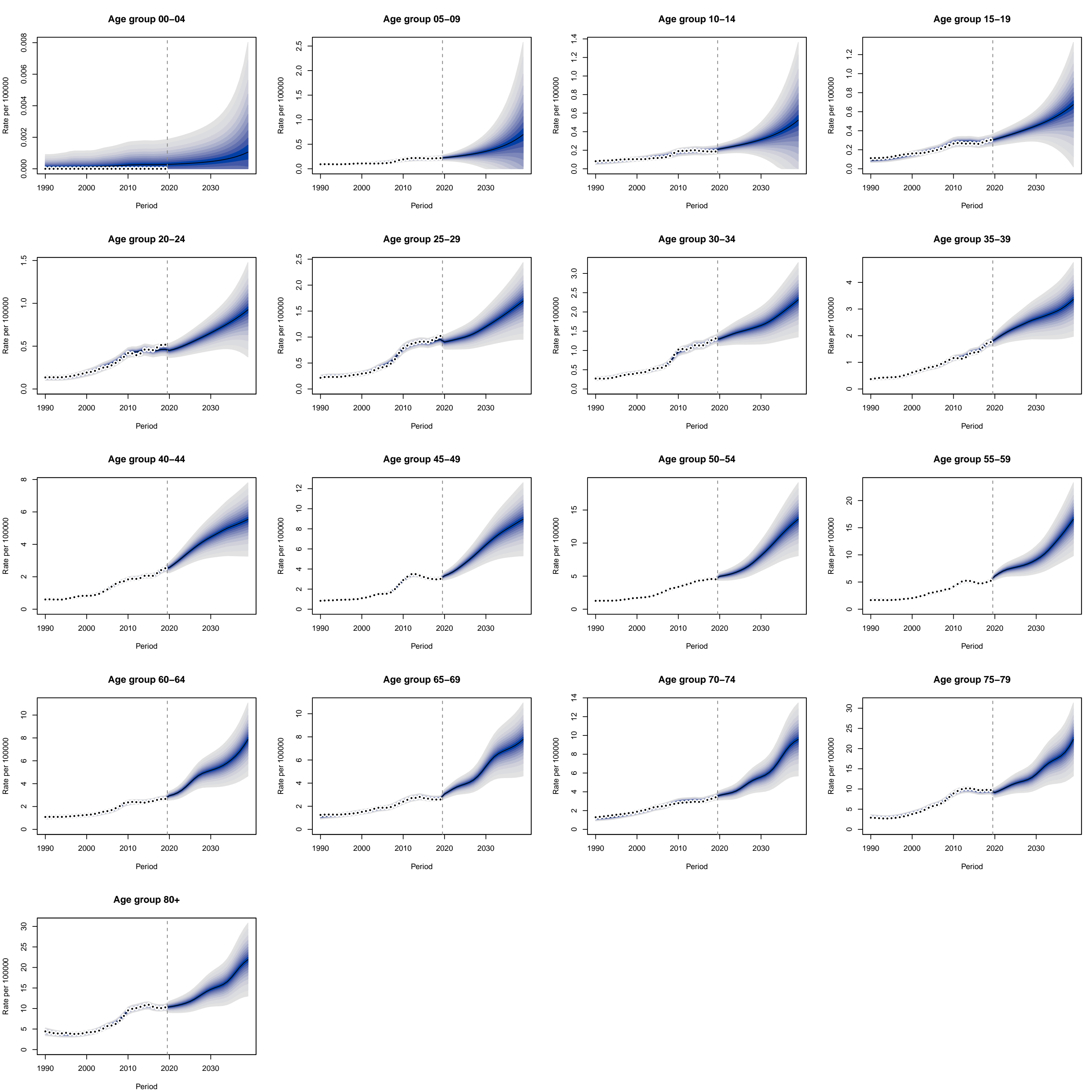

Supplement: Supplementary file 1 [file DataSheet_1.zip › Supplementary material_revised/figure S4 incidence rate of different age groups in males .pdf]

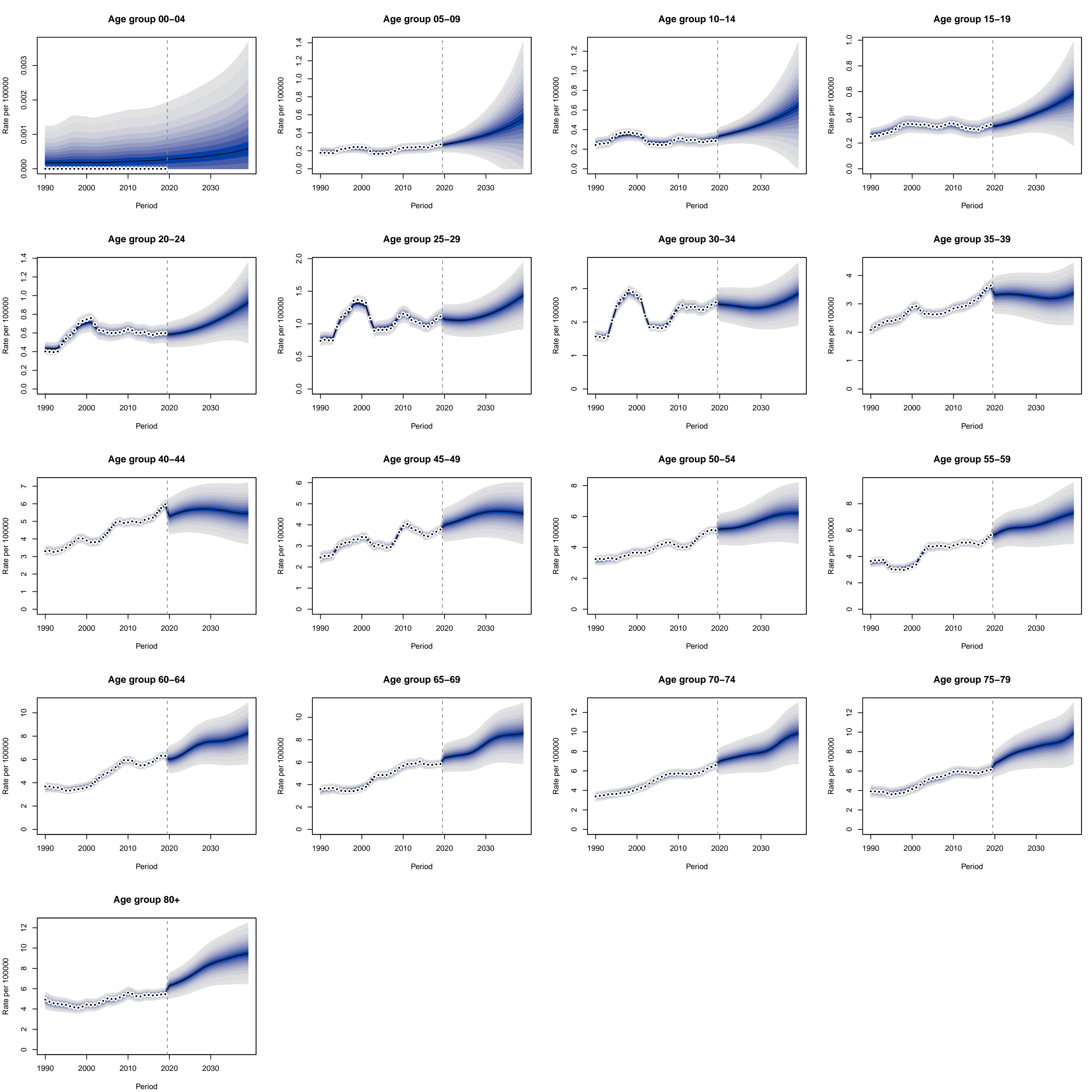

Supplement: Supplementary file 1 [file DataSheet_1.zip › Supplementary material_revised/figure S5 incidence rate of different age groups in females .pdf]

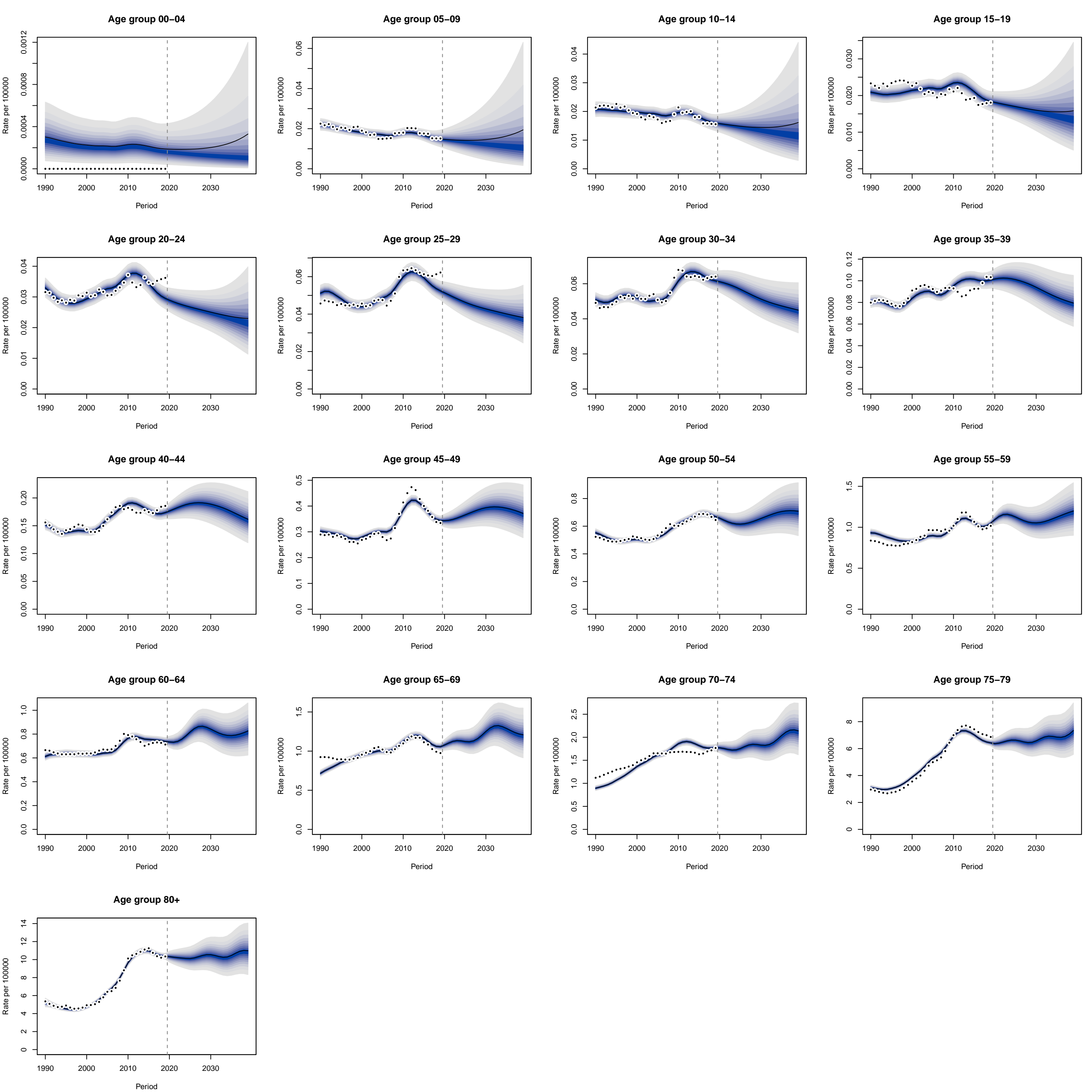

Supplement: Supplementary file 1 [file DataSheet_1.zip › Supplementary material_revised/figure S7 death rate of different age groups in males .pdf]
